# Supplementary material for: Improving the Obstetrics and Gynecology Learning Environment Through Faculty Development
Source: MedEdPORTAL. 2022 May 3;18:11246. doi: 10.15766/mep_2374-8265.11246 (PMC9061934; doi:10.15766/mep_2374-8265.11246)
Supplement: Supplementary file 1 — Preworkshop Survey.docxPowerPoint for the Learning Environment.pptxCases for the Learning Environment.docxFacilitator Guide.docxPostworkshop Survey.docx [file mep_2374-8265.11246-s001.zip › B. PowerPoint for the Learning Environment.pptx]

## Slide 1
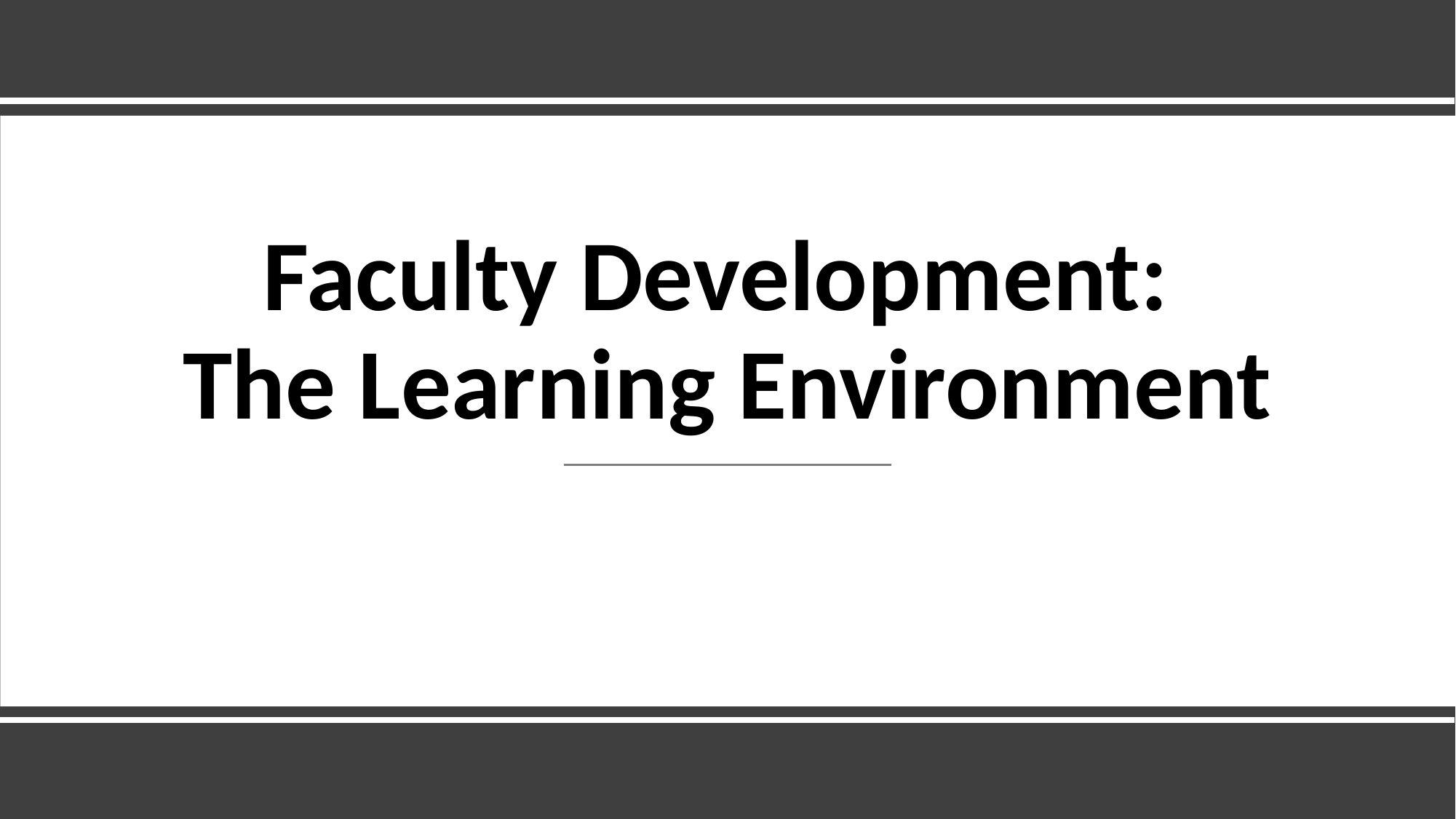

# Faculty Development:
The Learning Environment

## Slide 2
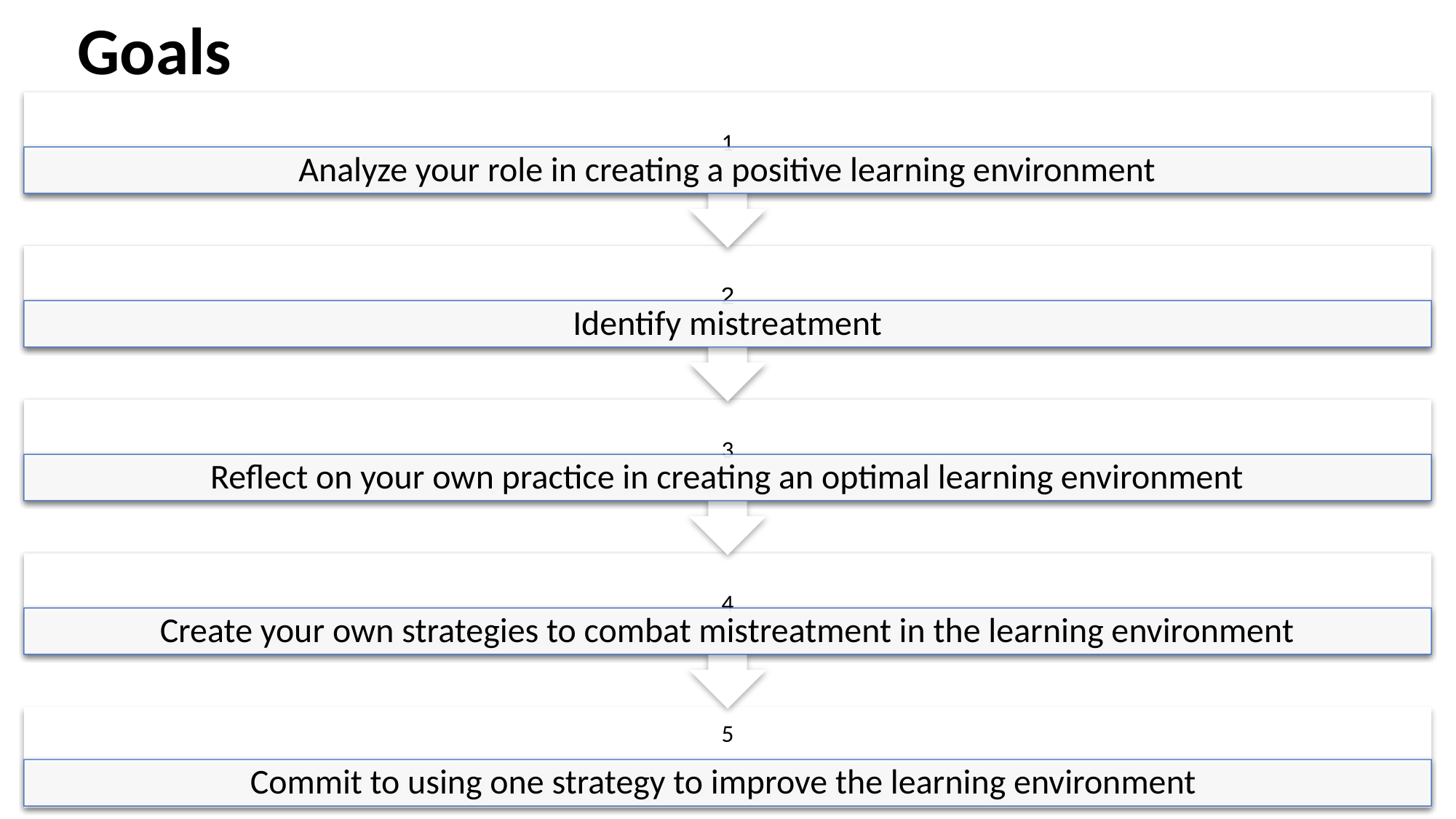

# Goals

## Slide 3
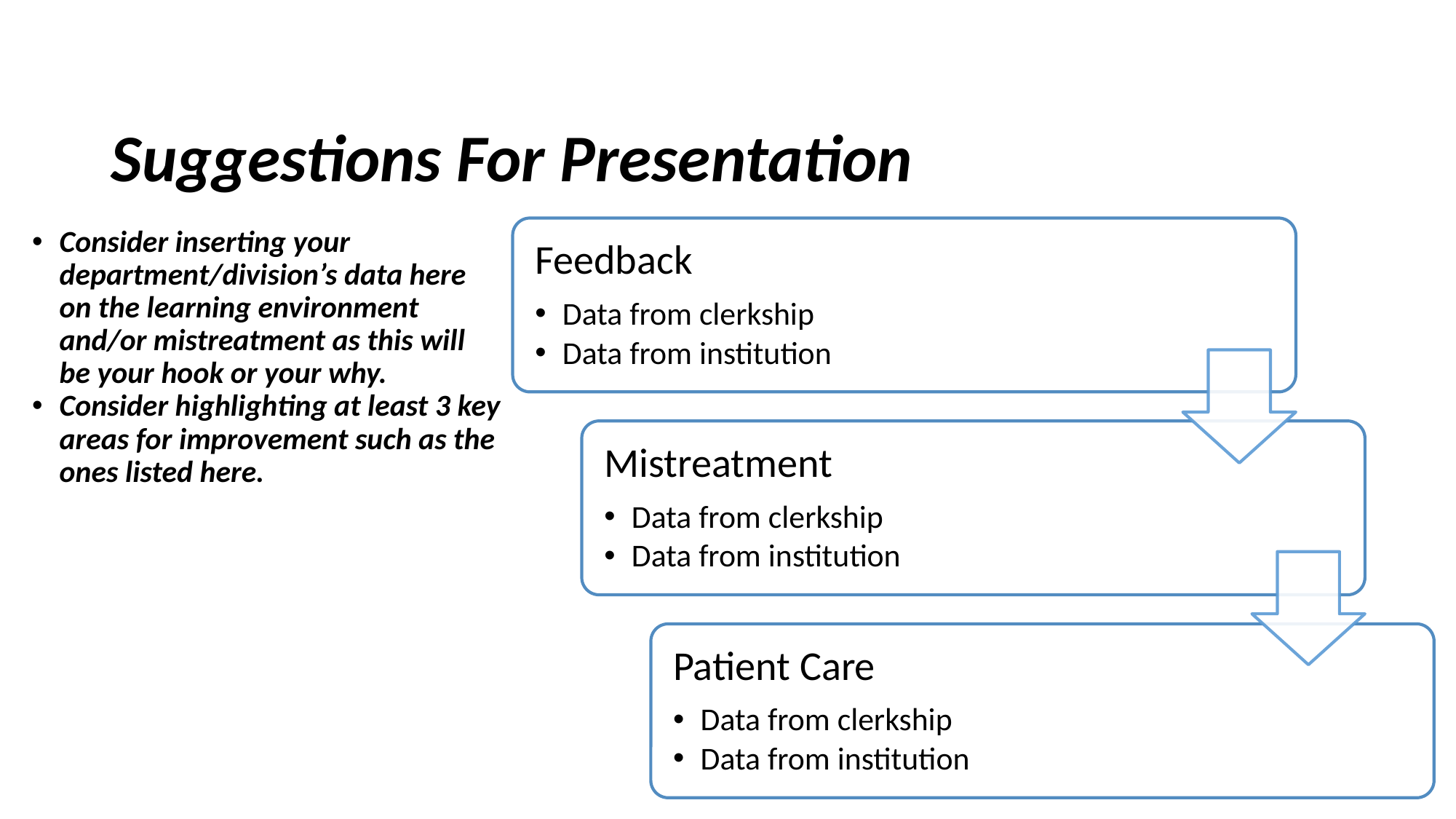

# Suggestions For Presentation
Consider inserting your department/division’s data here on the learning environment and/or mistreatment as this will be your hook or your why.
Consider highlighting at least 3 key areas for improvement such as the ones listed here.

## Slide 4
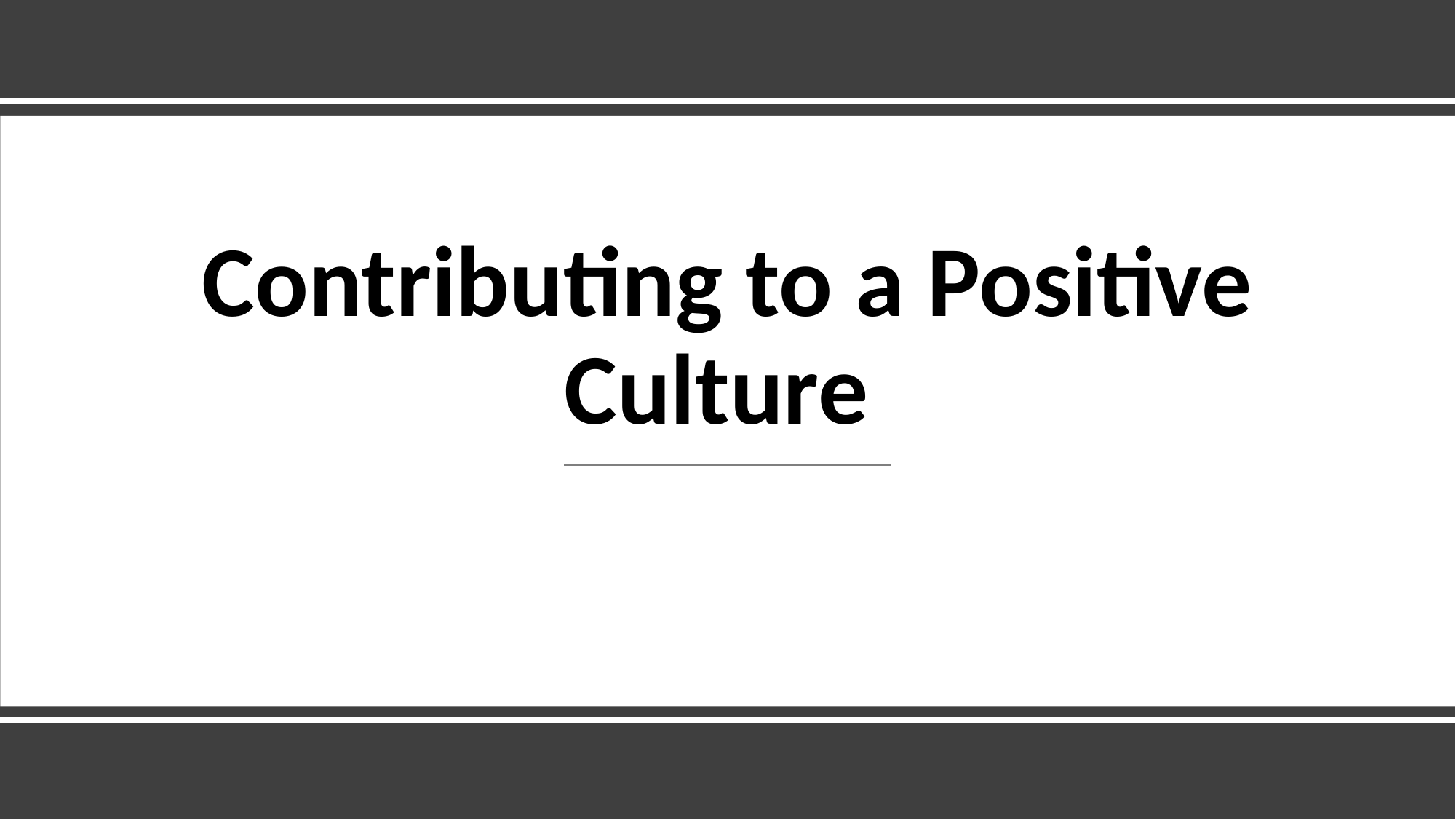

# Contributing to a Positive Culture

## Slide 5
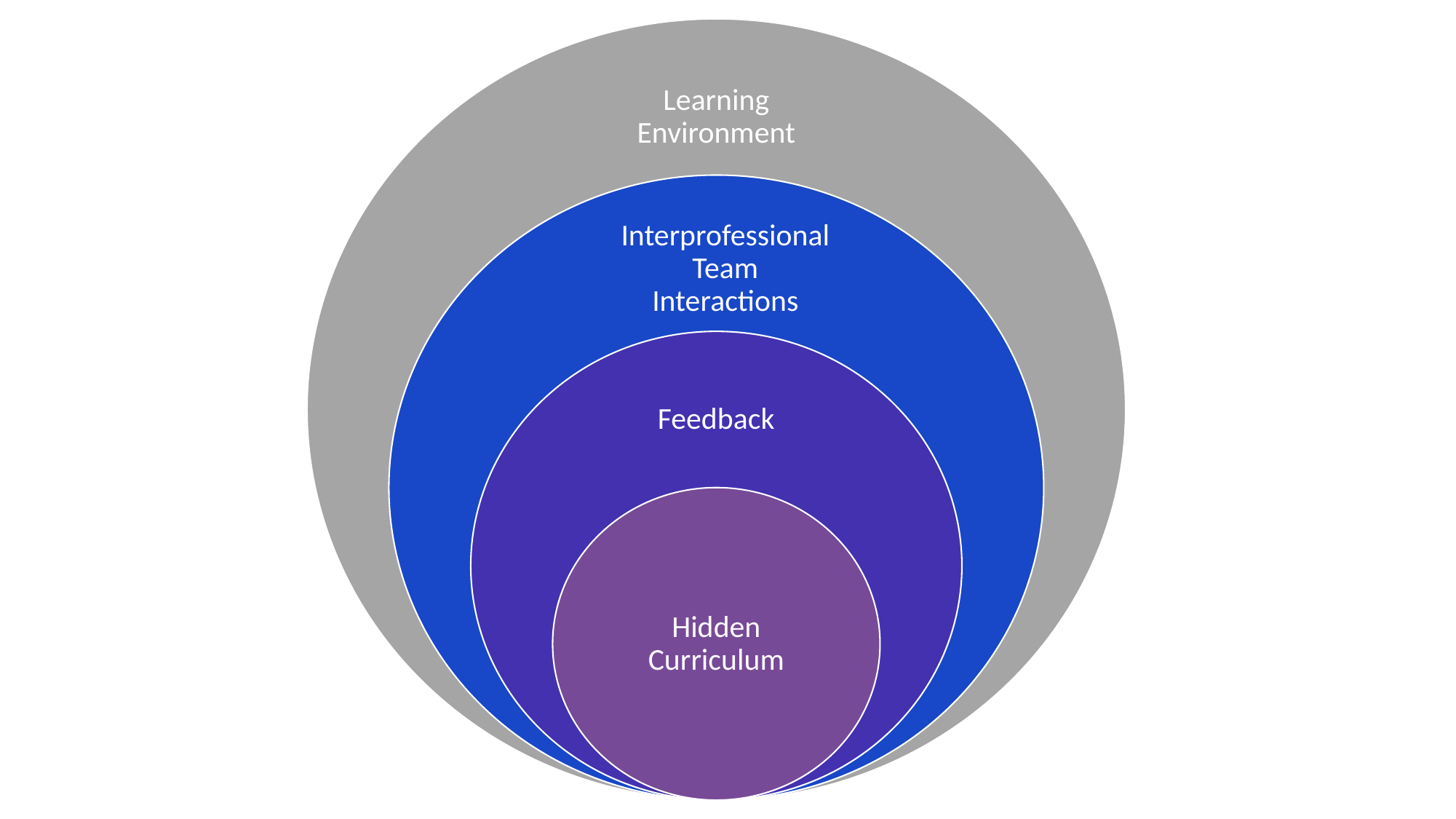

Learning Environment
Interprofessional Team Interactions
Feedback
Hidden Curriculum

## Slide 6
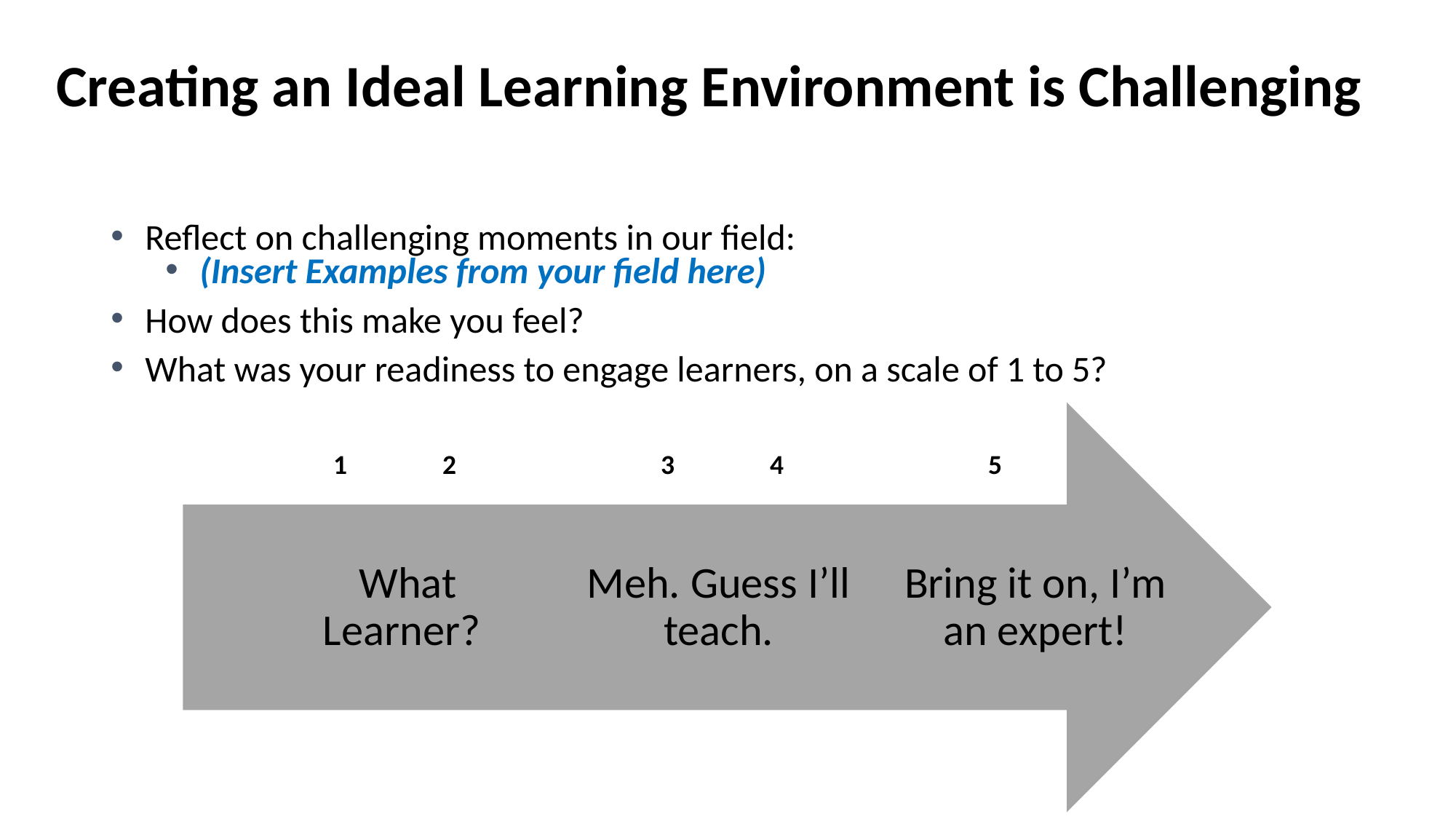

# Creating an Ideal Learning Environment is Challenging
Reflect on challenging moments in our field:
(Insert Examples from your field here)
How does this make you feel?
What was your readiness to engage learners, on a scale of 1 to 5?
 What Learner?
Meh. Guess I’ll teach.
Bring it on, I’m an expert!
1	2		3	4		5

## Slide 7
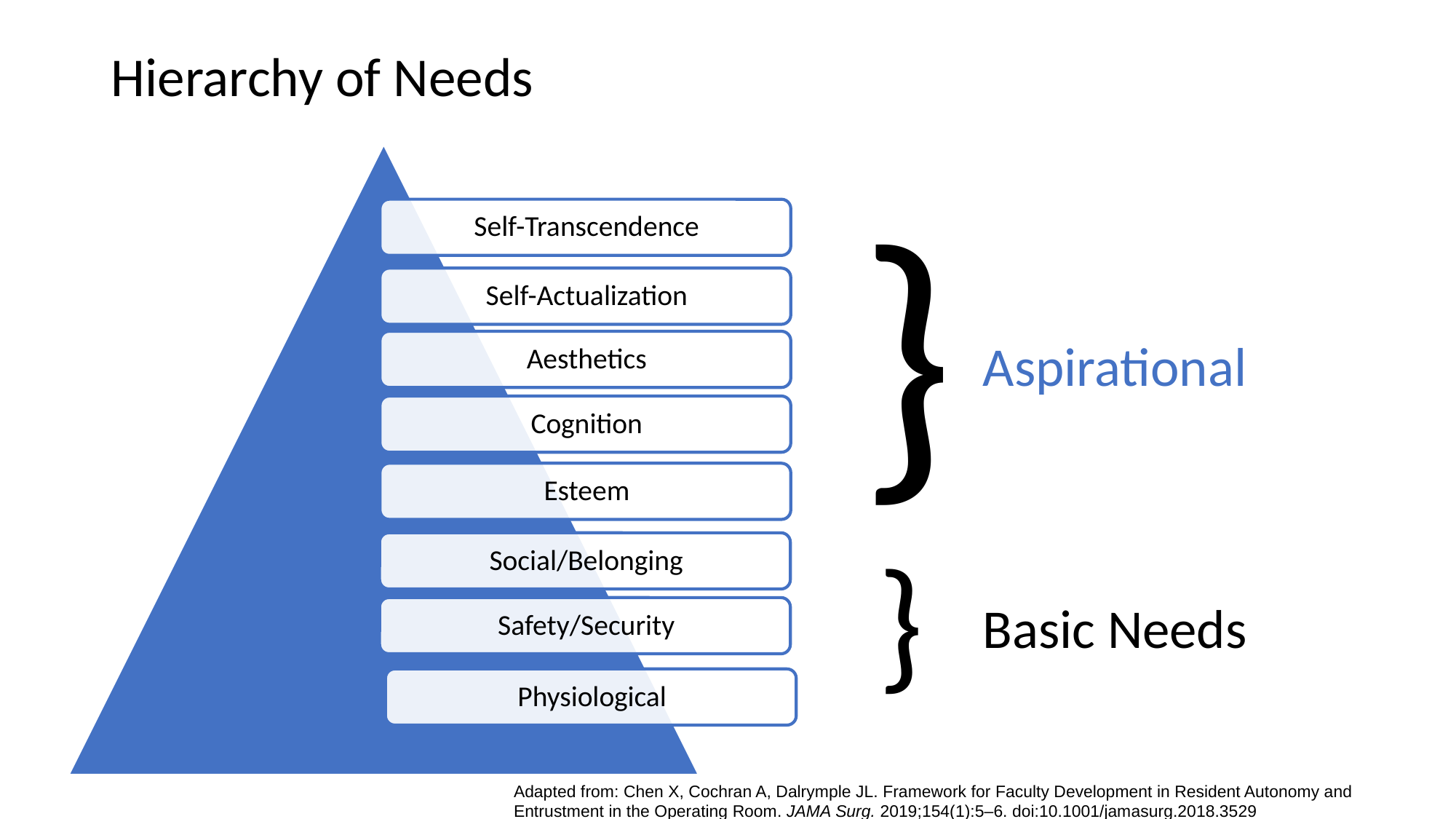

# Hierarchy of Needs
}
Aspirational
}
Basic Needs
Adapted from: Chen X, Cochran A, Dalrymple JL. Framework for Faculty Development in Resident Autonomy and Entrustment in the Operating Room. JAMA Surg. 2019;154(1):5–6. doi:10.1001/jamasurg.2018.3529

## Slide 8
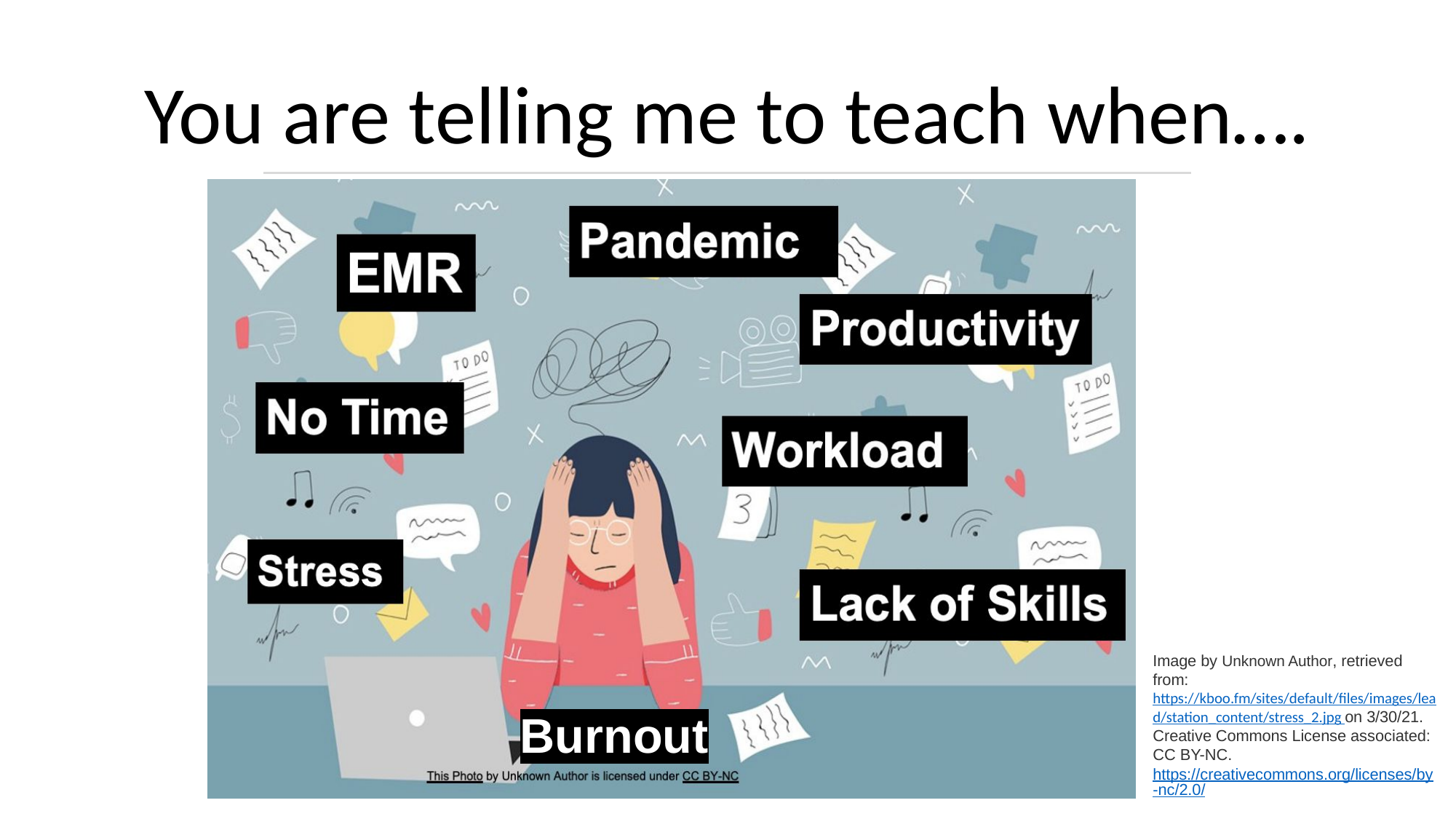

# You are telling me to teach when….
Pandemic
Image by Unknown Author, retrieved from: https://kboo.fm/sites/default/files/images/lead/station_content/stress_2.jpg on 3/30/21. Creative Commons License associated: CC BY-NC. https://creativecommons.org/licenses/by-nc/2.0/
Burnout

## Slide 9
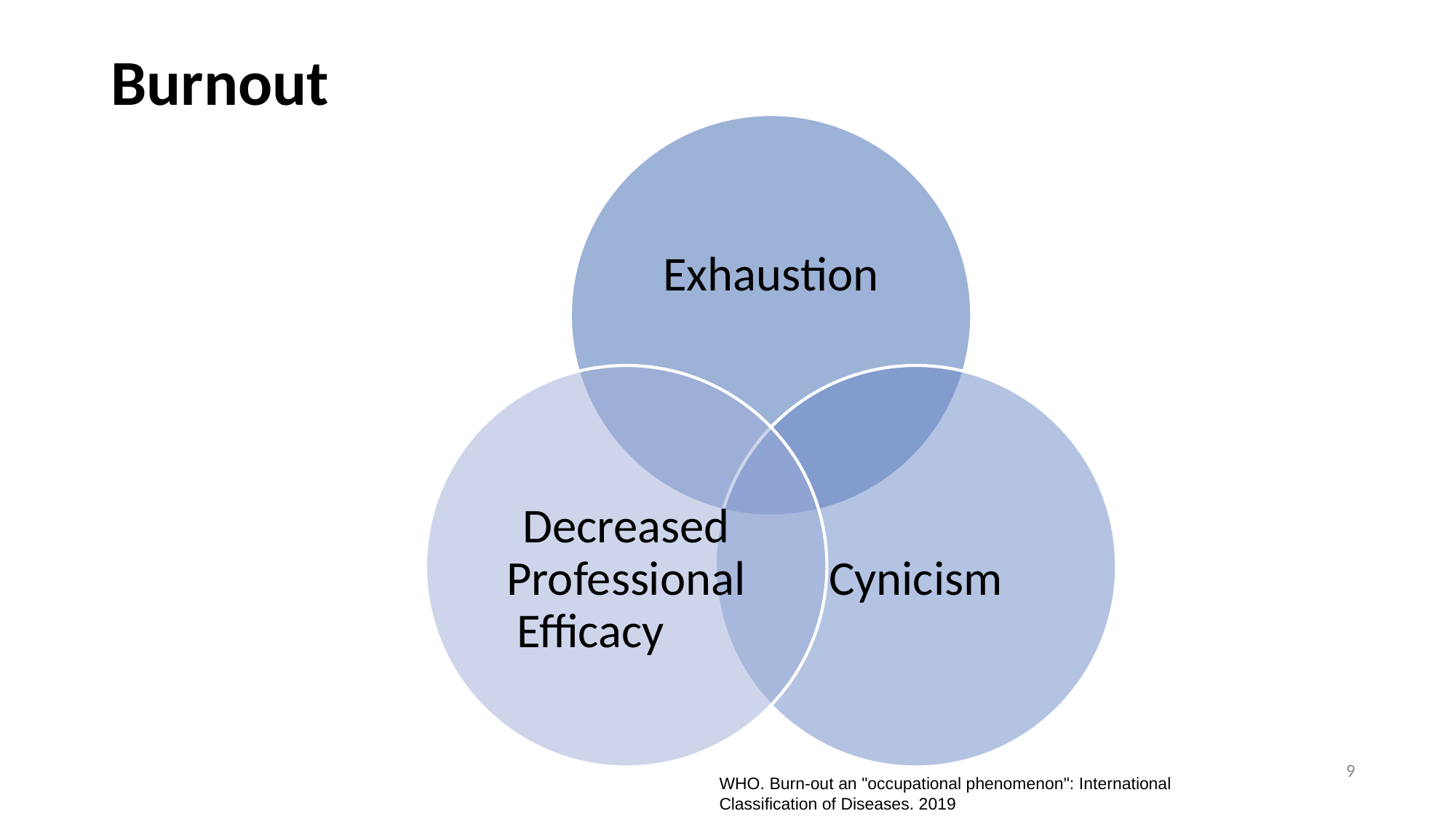

# Burnout
9
WHO. Burn-out an "occupational phenomenon": International Classification of Diseases. 2019

## Slide 10
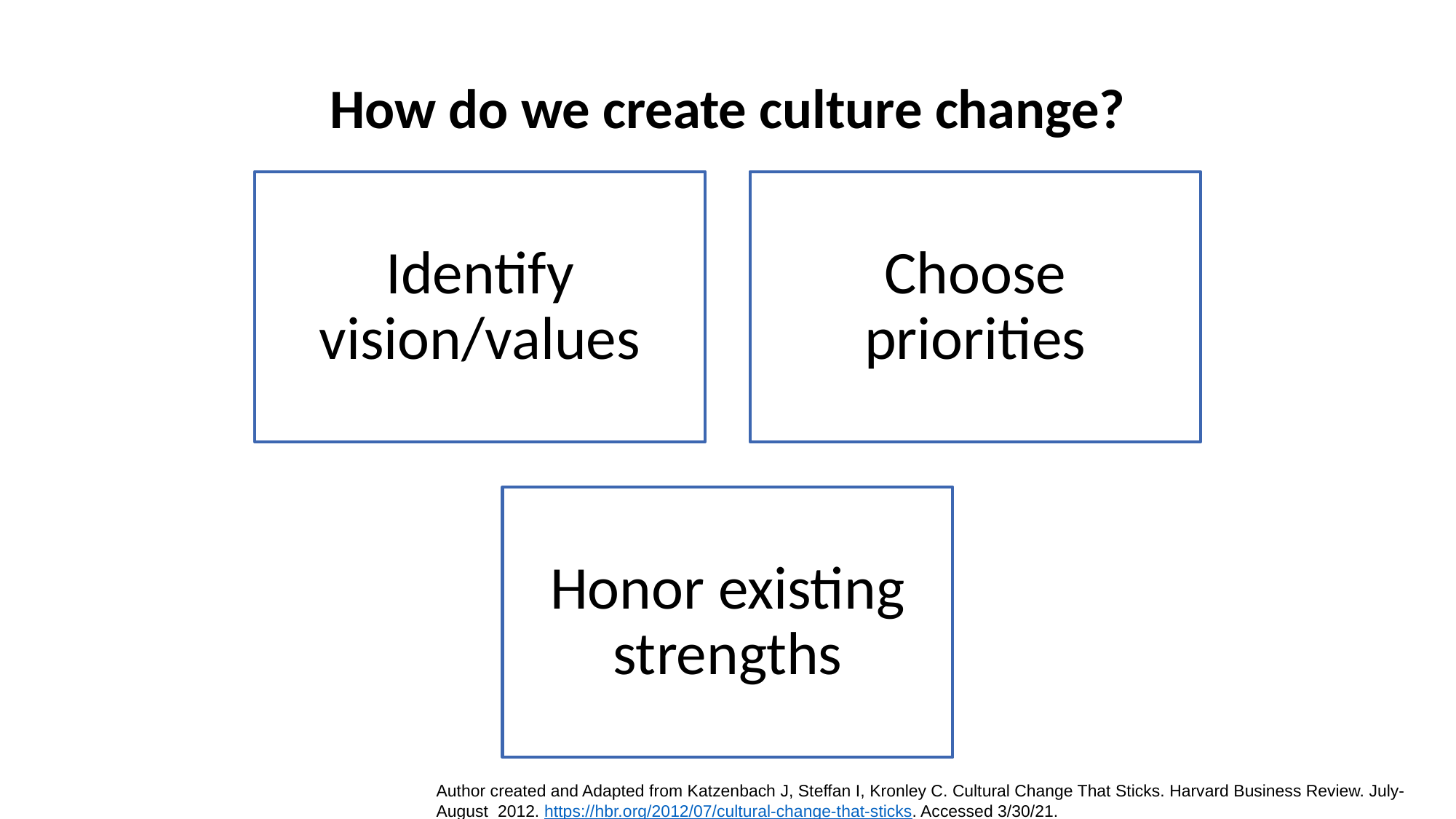

# How do we create culture change?
Author created and Adapted from Katzenbach J, Steffan I, Kronley C. Cultural Change That Sticks. Harvard Business Review. July-August 2012. https://hbr.org/2012/07/cultural-change-that-sticks. Accessed 3/30/21.

## Slide 11
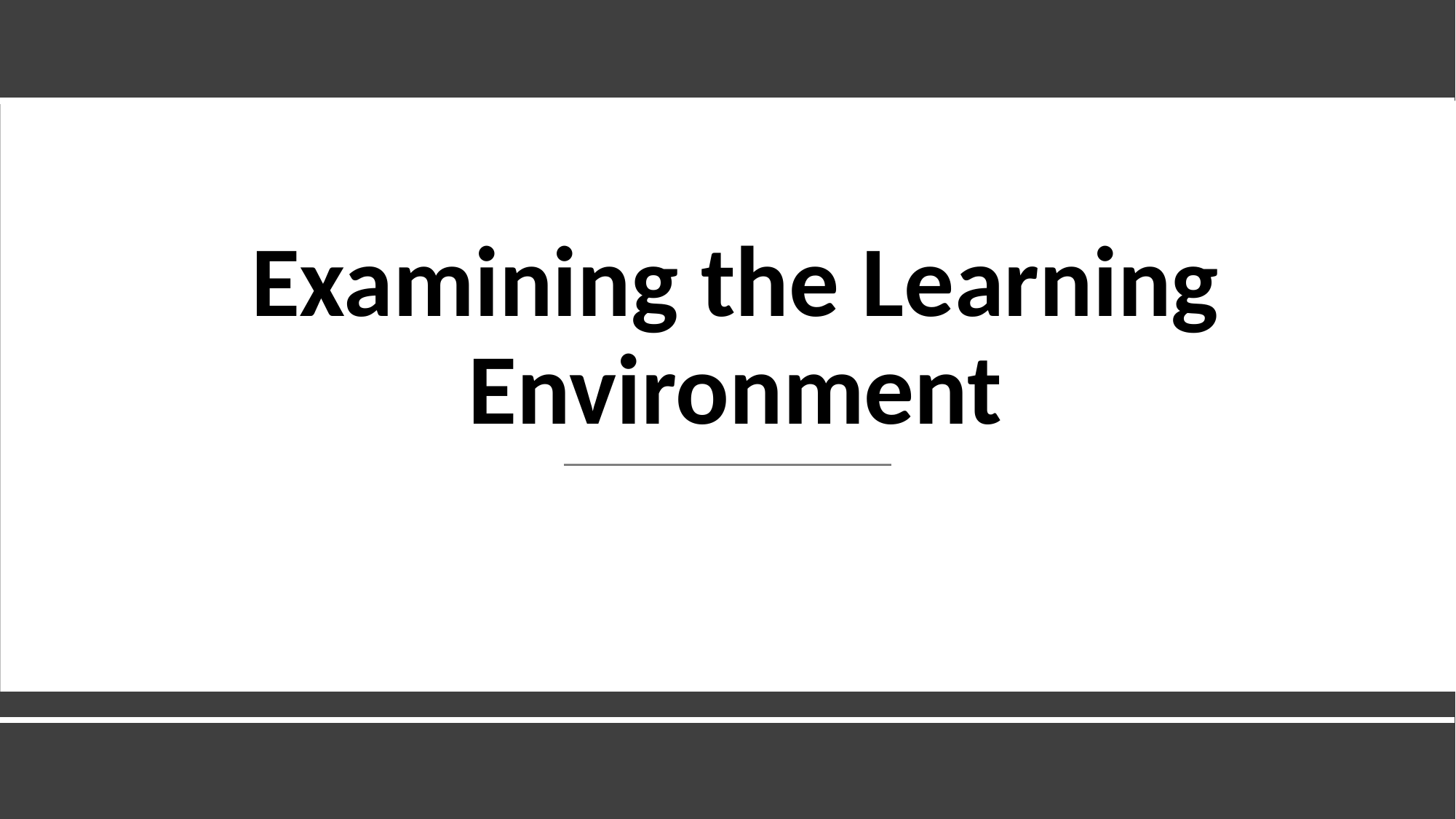

# Examining the Learning Environment

## Slide 12
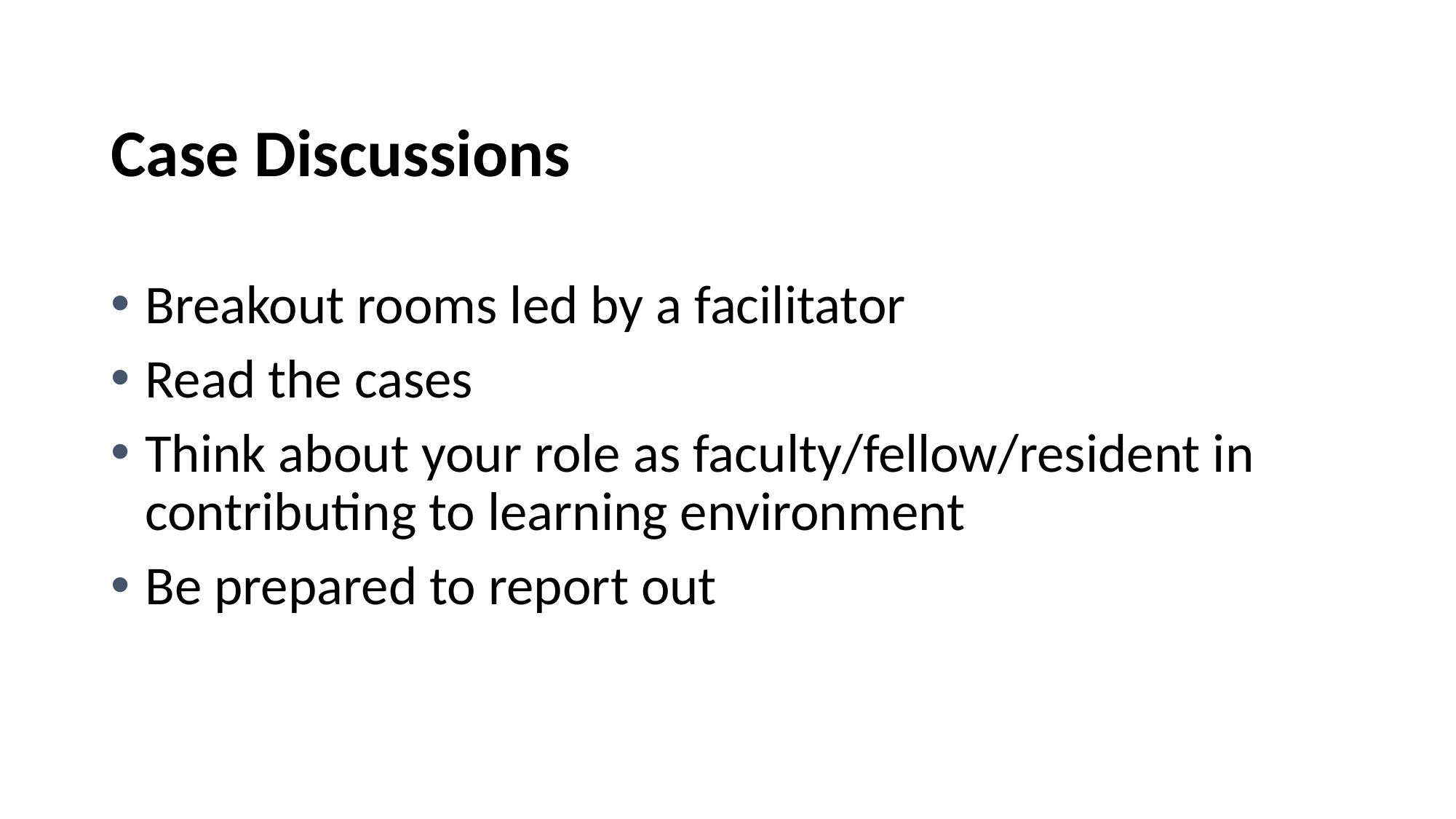

# Case Discussions
Breakout rooms led by a facilitator
Read the cases
Think about your role as faculty/fellow/resident in contributing to learning environment
Be prepared to report out

## Slide 13
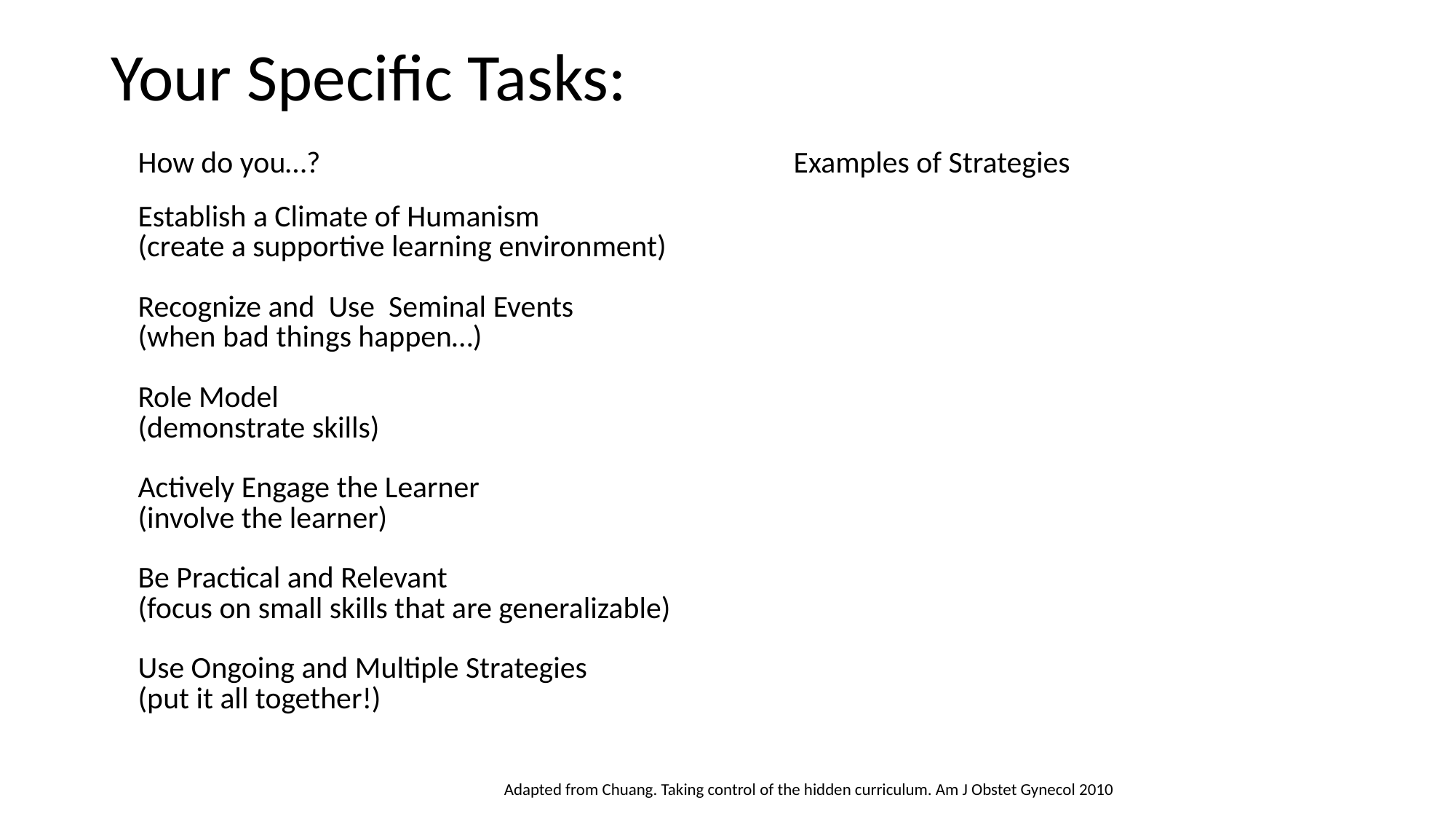

# Your Specific Tasks:
| How do you…? | Examples of Strategies |
| --- | --- |
| Establish a Climate of Humanism (create a supportive learning environment) | |
| Recognize and Use Seminal Events (when bad things happen…) | |
| Role Model (demonstrate skills) | |
| Actively Engage the Learner (involve the learner) | |
| Be Practical and Relevant (focus on small skills that are generalizable) | |
| Use Ongoing and Multiple Strategies (put it all together!) | |
Adapted from Chuang. Taking control of the hidden curriculum. Am J Obstet Gynecol 2010

## Slide 14
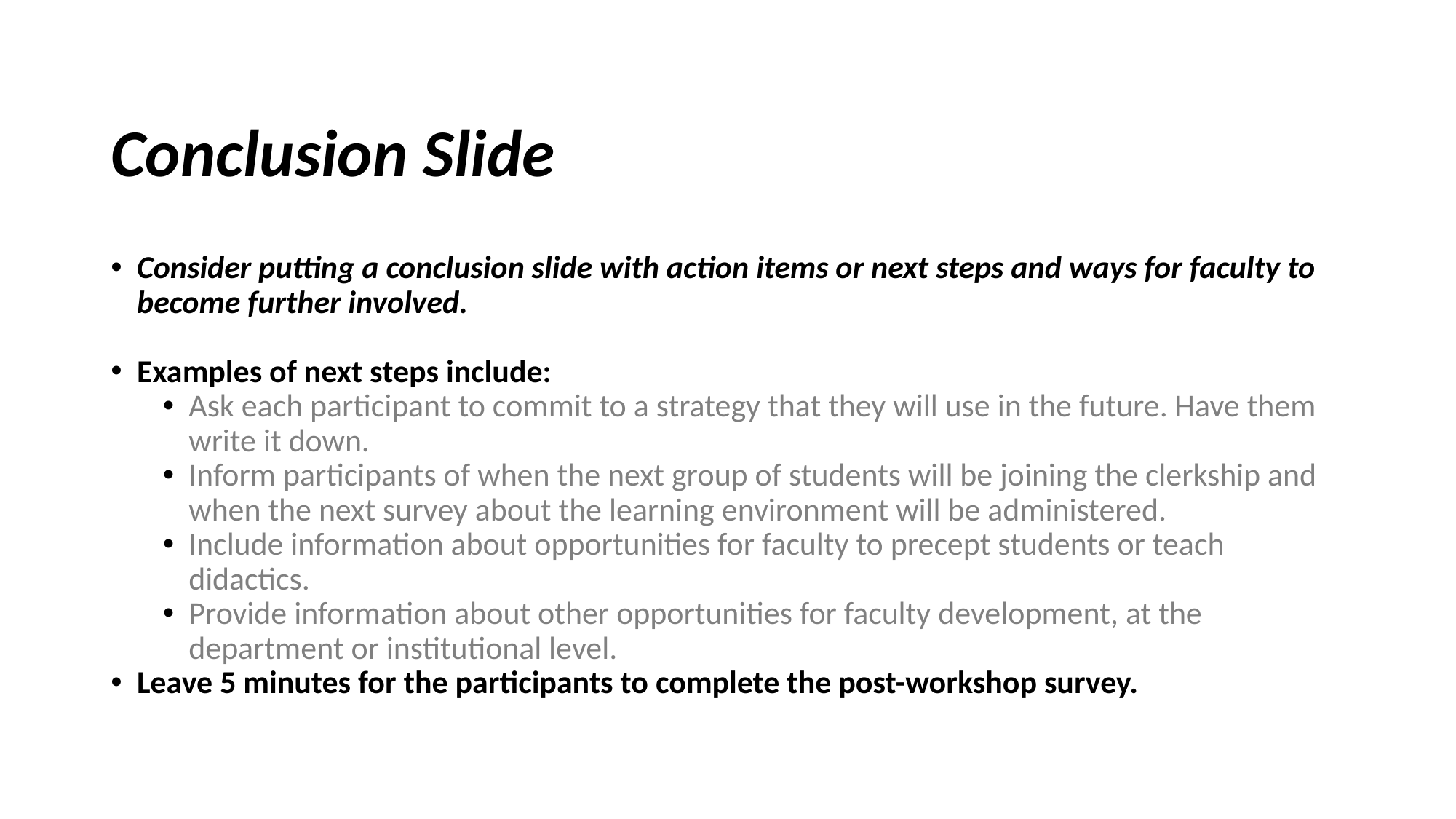

# Conclusion Slide
Consider putting a conclusion slide with action items or next steps and ways for faculty to become further involved.
Examples of next steps include:
Ask each participant to commit to a strategy that they will use in the future. Have them write it down.
Inform participants of when the next group of students will be joining the clerkship and when the next survey about the learning environment will be administered.
Include information about opportunities for faculty to precept students or teach didactics.
Provide information about other opportunities for faculty development, at the department or institutional level.
Leave 5 minutes for the participants to complete the post-workshop survey.
