# Supplementary material for: Improving the Obstetrics and Gynecology Learning Environment Through Faculty Development
Source: MedEdPORTAL. 2022 May 3;18:11246. doi: 10.15766/mep_2374-8265.11246 (PMC9061934; doi:10.15766/mep_2374-8265.11246)
Supplement: Supplementary file 1 — Preworkshop Survey.docxPowerPoint for the Learning Environment.pptxCases for the Learning Environment.docxFacilitator Guide.docxPostworkshop Survey.docx [file mep_2374-8265.11246-s001.zip › C. Cases for the Learning Environment.docx]

**Appendix C: Cases for the Learning Environment**

**Case 1:**

It is 3am, and the attending gets paged for a delivery. It has been a busy call night for the entire L&D team, with multiple deliveries. The attending, the chief resident, and the medical student walk into the room and get gowned and gloved. The patient is accompanied by her sisters and her mother. As the patient begins to push, the nurse asks the patient where the baby’s father is. After a brief pause, the patient responds quietly that the father is incarcerated. The nurse says, “that’s what I thought.” No one else responds. The attending remains silent throughout the delivery. The delivery is uncomplicated, and the attending leaves the room after the perineal laceration is repaired.

| **Examples of Strategies** |
| --- |
|  |
|  |

**Case 2:**

It is a busy day in the clinic, and the attending, Dr. Jones, has 22 patients on his schedule. Dr. Jones sees the first patient, and as he is leaving the room, he asks the medical assistant for the next patient’s vital signs. The medical assistant states that a medical student already started seeing the second patient. Dr. Jones was not expecting a learner in his clinic today. At that moment, the medical student, John, comes out of the patient’s room and starts presenting the patient to Dr. Jones. Dr. Jones holds up a hand and says, “Stop right there. You didn’t introduce yourself to me and didn’t ask for permission to see the patient.” John apologizes and introduces himself, stating that the clerkship director specifically assigned him to the clinic today because of his interest in primary care. He then continues with the presentation of a 55-year-old woman with a new diagnosis of hypertension. Dr. Jones asks John the recommended treatment of hypertension in a patient with diabetes, and John does not know the answer.

| \| **Examples of Strategies** \| \| --- \| \|  \| \|  \| |  |
| --- | --- | --- | --- | --- |

**Case 3:**

The intern is managing a busy service and has worked nearly 80 hours this week alone. The team had ordered a cardiology consult at 7am on a medically complex patient awaiting surgery. It is now 4pm, and the cardiology team has not yet seen the patient; the surgery cannot proceed until cardiology has evaluated the patient. The intern urgently pages the cardiology team, who then evaluates the patient and provides recommendations. The intern puts in the required orders for the patient. She updates the team census, preps the patient for surgery the next day by making the patient NPO, and gives signout to the overnight team. She leaves the hospital, and as she is leaving, realizes that she did not update the fellow, who is still in the OR, about the patient. She shrugs it off, thinking that because she completed the tasks, there is no need to let the fellow know, and she really wants to go home before 6:30pm one day this week. While she is on her way home, the fellow calls the intern and chastises her for not giving the fellow signout. The intern apologizes and starts to cry. The fellow hangs up. Later that week, the intern overhears the fellow stating that “this is the worst intern ever!”

| **Examples of Strategies** |
| --- |

**Case 4:**

The chief resident is 19 hours into her second 24-hour shift in 9 days. The junior presents a patient to her who may need methotrexate for a possible ectopic pregnancy. The preliminary read on the ultrasound shows no intrauterine gestational sac and with an hcg level is 2000 mlU/ml. The junior resident reports the patient’s prior hcg values and the chief calculates that there has been an inappropriate rise in the hcg level. She tells the resident to give the patient methotrexate. The next day, the attending reviews the final read of the ultrasound and calls the chief resident because the final read shows an intrauterine gestational sac with no fetal pole or yolk sac. The attending informs the chief resident that her calculations were incorrect, and that there was, in fact, an appropriate rise in the hcg level. The chief resident is noticeably upset over the phone and apologizes profusely, but the attending remains silent and then hangs up the phone. The chief resident calls the junior to check in on her. The junior resident is also upset, and the chief apologizes to the junior. The attending does not call either of them again and does not debrief the situation.

| **Examples of Strategies** |
| --- |

|  |
| --- |
|  |
