# Supplementary material for: Improving the Obstetrics and Gynecology Learning Environment Through Faculty Development
Source: MedEdPORTAL. 2022 May 3;18:11246. doi: 10.15766/mep_2374-8265.11246 (PMC9061934; doi:10.15766/mep_2374-8265.11246)
Supplement: Supplementary file 1 — Preworkshop Survey.docxPowerPoint for the Learning Environment.pptxCases for the Learning Environment.docxFacilitator Guide.docxPostworkshop Survey.docx [file mep_2374-8265.11246-s001.zip › D. Facilitator Guide.docx]

**Appendix D: Facilitator Guide**

***Each facilitator(s) has their group read the assigned case and leads the group through the discussion questions. Facilitators can utilize the chart below to take notes on strategies.***

**Case 1: *Establish a Climate of Humanism; Role Model***

It is 3am, and the attending gets paged for a delivery. It has been a busy call night for the entire L&D team, with multiple deliveries. The attending, the chief resident, and the medical student walk into the room and get gowned and gloved. The patient is accompanied by her sisters and her mother. As the patient begins to push, the nurse asks the patient where the baby’s father is. After a brief pause, the patient responds quietly that the father is incarcerated. The nurse says, “that’s what I thought.” No one else responds. The attending remains silent throughout the delivery. The delivery is uncomplicated, and the attending leaves the room after the perineal laceration is repaired.

**Discussion Questions for the Group:**

- How did this scenario make you feel? What was your gut reaction?
- What do people think about the nurse’s comments?
- If you were present in the room, what would you have done differently?
- How might instances like this contribute to the learning environment?
- What is one take away that you have for how to establish a climate of humanism?
- What is one take away that you have for how to role model?

·

·

·

| **How do you…?** | **Examples of Strategies** |
| --- | --- |
| Establish a Climate of Humanism  (create a supportive learning environment) |  |
| Role Model  (demonstrate skills) |  |

**Case 2: *Actively engage the learner; be practical and relevant***

It is a busy day in the clinic, and the attending, Dr. Jones, has 22 patients on his schedule. Dr. Jones sees the first patient, and as he is leaving the room, he asks the medical assistant for the next patient’s vital signs. The medical assistant states that a medical student already started seeing the second patient. Dr. Jones was not expecting a learner in his clinic today. At that moment, the medical student, John, comes out of the patient’s room and starts presenting the patient to Dr. Jones. Dr. Jones holds up a hand and says, “Stop right there. You didn’t introduce yourself to me and didn’t ask for permission to see the patient.” John apologizes and introduces himself, stating that the clerkship director specifically assigned him to the clinic today because of his interest in primary care. He then continues with the presentation of a 55-year-old woman with a new diagnosis of hypertension. Dr. Jones asks John the recommended treatment of hypertension in a patient with diabetes, and John does not know the answer.

**Discussion Questions:**

- What is the learner’s responsibility?
- What is the attending’s responsibility?
- How might Dr. Jones have reacted to create a more positive or welcoming learning environment?
- How might instances like this contribute to the learning environment?
- What is one take away that you have for how to actively engage the learner?
- What is one take away that you have for how to be practical and relevant?

| **How do you…?** | **Examples of Strategies** |
| --- | --- |

| Actively Engage the Learner  (involve the learner) |  |
| --- | --- |
| Be Practical and Relevant  (focus on small skills that are generalizable) |  |

**Case 3: *Actively engage the learner; be practical and relevant***

The intern is managing a busy service and has worked nearly 80 hours this week alone. The team had ordered a cardiology consult at 7am on a medically complex patient awaiting surgery. It is now 4pm, and the cardiology team has not yet seen the patient; the surgery cannot proceed until cardiology has evaluated the patient. The intern urgently pages the cardiology team, who then evaluates the patient and provides recommendations. The intern puts in the required orders for the patient. She updates the team census, preps the patient for surgery the next day by making the patient NPO, and gives signout to the overnight team. She leaves the hospital, and as she is leaving, realizes that she did not update the fellow, who is still in the OR, about the patient. She shrugs it off, thinking that because she completed the tasks, there is no need to let the fellow know, and she really wants to go home before 6:30pm one day this week. While she is on her way home, the fellow calls the intern and chastises her for not giving the fellow signout. The intern apologizes and starts to cry. The fellow hangs up. Later that week, the intern overhears the fellow stating that “this is the worst intern ever!”

**Discussion Questions:**

- What do you think the intern was experiencing?
- What do you think the fellow was experiencing?
- How could the fellow have provided this feedback in a different way?
- How might instances like this contribute to the learning environment?
- What is one take away that you have for how to actively engage the learner?
- What is one take away that you have for how to be practical and relevant?

| **How do you…?** | **Examples of Strategies** |
| --- | --- |

| Actively Engage the Learner  (involve the learner) |  |
| --- | --- |
| Be Practical and Relevant  (focus on small skills that are generalizable) |  |

**Case 4: *Recognize and use seminal events; role model***

The chief resident is 19 hours into her second 24-hour shift in 9 days. The junior presents a patient to her who may need methotrexate for a possible ectopic pregnancy. The preliminary read on the ultrasound shows no intrauterine gestational sac and with an hcg level is 2000 mlU/ml. The junior resident reports the patient’s prior hcg values and the chief calculates that there has been an inappropriate rise in the hcg level. She tells the resident to give the patient methotrexate. The next day, the attending reviews the final read of the ultrasound and calls the chief resident because the final read shows an intrauterine gestational sac with no fetal pole or yolk sac. The attending informs the chief resident that her calculations were incorrect, and that there was, in fact, an appropriate rise in the hcg level. The chief resident is noticeably upset over the phone and apologizes profusely, but the attending remains silent and then hangs up the phone. The chief resident calls the junior to check in on her. The junior resident is also upset, and the chief apologizes to the junior. The attending does not call either of them again and does not debrief the situation.

**Discussion Questions:**

- If you were the program director, how might you give the chief resident feedback? What did she do that was right? Where might she have improved?
- What could the attending have done differently? How could the attending have prepared the team to provide this information to the patient?
- When medical errors occur, how can we provide feedback in a constructive manner?
- How might instances like this contribute to the learning environment?
- What is one take away that you have for how to recognize and use seminal events?
- What is one take away that you have for how to role model?

| **How do you…?** | **Examples of Strategies** |
| --- | --- |

| Recognize and Use Seminal Events  (when bad things happen…) |  |
| --- | --- |
| Role Model  (demonstrate skills) |  |

**GENERAL TIPS FOR FACILITATORS:**

*Keeping the conversation on task:*

- Set a clear goal that the group will be delivering a set of strategies and that the entire group will contribute to the development of these strategies.
- If a participant has deviated from the discussion questions, thank them for their thoughts, then ask the rest of the group what their thoughts are about a specific aspect of the case.
- If one participant is dominating the conversation, ask other members what their thoughts are about what the first participant shared.

*Breaking the silence:*

- Have someone in the group read the case out loud.
- If your group is quiet, give everyone 30 seconds to 1 minute to think, and then ask again if there are any volunteers to open the discussion.
- If you do “cold call” on someone, ask a broad question so that the person can share any aspects of the case that resonates with them.
- Ask open ended questions to encourage discussion.
- Ask follow up questions to prompt discussion about another participant’s response.
- If there truly is silence, then consider changing the scenario slightly to create a scenario that might be more familiar to participants.

*The authors acknowledge that while these cases may be specific to obstetrics and gynecology, the conflicts and/or issues that arise in each case are universally applicable across specialties. If colleagues would like to adapt the cases for their particular specialty, we have included below the key aspects of each case needed to illustrate the conflicts/issues. Colleagues can adapt each of these core issues and incorporate them into timely and realistic examples from their own specialty.*

Case 1:

Key Aspect:

- Faculty member witnesses a microaggression but does not address it with his or her team, and does not speak to the perpetrator at all.

Case 2:

Key Aspect:

- The attending feels that the learner has not adequately prepared for the clinical scenario and gives that feedback in a harsh manner.

Case 3:

Key Aspect:

- The intern forgets to update the fellow leading the team about critical developments with a patient; the fellow provides the intern feedback in a harsh manner and criticizes her to other team members.

Case 4:

Key Aspect:

- A medical error occurs, and the attending does not debrief with the team or the patient.
