# Supplementary material for: Improving the Obstetrics and Gynecology Learning Environment Through Faculty Development
Source: MedEdPORTAL. 2022 May 3;18:11246. doi: 10.15766/mep_2374-8265.11246 (PMC9061934; doi:10.15766/mep_2374-8265.11246)
Supplement: Supplementary file 1 — Preworkshop Survey.docxPowerPoint for the Learning Environment.pptxCases for the Learning Environment.docxFacilitator Guide.docxPostworkshop Survey.docx [file mep_2374-8265.11246-s001.zip › E. Postworkshop Survey.docx]

**Appendix E: Post-Workshop Survey:**

1. Please respond to the statements below:

|  | Strongly Agree | Agree | Neutral | Disagree | Strongly Disagree | Not Applicable |
| --- | --- | --- | --- | --- | --- | --- |
| I know what my role is as a faculty member in contributing to the learning environment |  |  |  |  |  |  |
| I feel well prepared to engage medical students when I am the assigned faculty preceptor |  |  |  |  |  |  |
| I feel well prepared to engage residents when I am the assigned faculty preceptor |  |  |  |  |  |  |
| I have the skills needed to be an effective teacher |  |  |  |  |  |  |
| I consistently try to use effective teaching strategies |  |  |  |  |  |  |
| I aim to create an inclusive environment when teaching |  |  |  |  |  |  |
| I routinely involve medical students in clinical care of patients |  |  |  |  |  |  |
| I routinely involve residents in clinical care of patients |  |  |  |  |  |  |
| I am more aware of the issues around the learning environment after today’s presentation |  |  |  |  |  |  |
| I feel that my interaction with students will change in a positive way based on today’s discussion |  |  |  |  |  |  |
| I feel that my interaction with residents will change in a positive way based on today’s discussion |  |  |  |  |  |  |
| I am more inclined to create an inclusive learning environment after today’s presentation |  |  |  |  |  |  |
| This faculty development Grand Rounds was relevant to my needs |  |  |  |  |  |  |
| I learned a new skill during this Grand Rounds |  |  |  |  |  |  |

1. Name 1 strategy you will commit to using in order to contribute to a positive learning environment:
2. What is your age?

_____

1. What is your gender identity?

Male

Female

Non-binary

Prefer not to disclose

1. What is your race/ethnicity?

Hispanic

Black

Caucasian

Asian

American Indian/Pacific Islander

Prefer not to disclose

1. How many years have you been here at the BIDMC?
