# Supplementary material for: Improving the Obstetrics and Gynecology Learning Environment Through Faculty Development
Source: MedEdPORTAL. 2022 May 3;18:11246. doi: 10.15766/mep_2374-8265.11246 (PMC9061934; doi:10.15766/mep_2374-8265.11246)
Supplement: Supplementary file 1 — Preworkshop Survey.docxPowerPoint for the Learning Environment.pptxCases for the Learning Environment.docxFacilitator Guide.docxPostworkshop Survey.docx [file mep_2374-8265.11246-s001.zip › Educational_Summary_Report_11246.pdf]

MedEdPORTAL® The AAMC Journal of Teaching and Learning Resources

Original Publication Open Access

Improving the Obstetrics and Gynecology Learning Environment Through Faculty Development

Huma Farid, MD*, John L. Dalrymple, MD, Monica Mendiola, MD, Celeste Royce, MD, Brett Young, MD, Katharyn Meredith Atkins, MD

*Corresponding author: hfarid@bidmc.harvard.edu

Abstract

Introduction: The learning environment is shaped by both formal and hidden curricula. Faculty play a critical role in the learning environment but may not be prepared to address the hidden curriculum. This workshop teaches faculty how to manage the hidden curriculum's challenges. **Methods:** Medical students' end-of-clerkship evaluations revealed low ratings in the domains of feedback, respectful interactions, professional language use, and empathy. We created a virtual 60-minute case-based faculty development workshop to highlight the role of faculty in improving the learning environment. A preworkshop survey was emailed to participants. At the workshop, following a brief introduction, participants were divided into groups to discuss the cases and develop strategies to improve the learning environment. A postworkshop survey was used to assess the workshop. **Results:** Sixty faculty members attended the seminar. Fifty-seven percent completed a preworkshop survey, and 33% completed the postworkshop survey. After the workshop, more faculty felt well prepared to engage students and residents. The majority of participants (85%) reported being more aware of issues around the learning environment. Most (85%) felt that their interactions with medical students would change in a positive way after the workshop. Ninety percent agreed the workshop was relevant to their needs, 70% agreed they learned a new skill in the workshop, and 80% committed to creating an inclusive learning environment after the workshop. **Discussion:** This workshop was well received by participants and was associated with an improvement in learning environment ratings. Faculty development seminars are an efficient tool to improve the learning environment.

Keywords
Hidden Curriculum, Mistreatment, Case-Based Learning, Clinical Teaching/Bedside Teaching, Faculty Development, Learning Environment

Educational Objectives

By the end of this activity, learners will be able to:

1. Analyze their role in creating a positive learning environment.
2. Identify mistreatment and aspects of a suboptimal learning environment by critiquing cases.
3. Reflect on their own practice in creating an optimal learning environment.
4. Create their own strategies to combat mistreatment in the learning environment.
5. Commit to routinely incorporating at least one strategy into their teaching repertoire.

Citation:
Farid H, Dalrymple JL, Mendiola M, Royce C, Young B, Atkins KM. Improving the obstetrics and gynecology learning environment through faculty development. *MedEdPORTAL*. 2022;18:11246.
https://doi.org/10.15766/mep_2374-8265.11246

Introduction

The clinical learning environment is shaped by multiple factors, including the formal curriculum; interactions with faculty, residents, staff, and peers; and other aspects of the hidden curriculum,[1] which is defined as the "set of influences that function at the level of organizational structure and culture."[2] The learning environment impacts students' ability to learn,[3] the depth and breadth of their clinical experiences, student wellness,[4] and academic achievement and satisfaction.[1] Experiences of mistreatment among medical students[5,6] reflect the health of the learning environment. Unfortunately, mistreatment is common, with up to 83% of students reporting at least one experience of mistreatment by residents, faculty, or staff.[6] These experiences range from microaggressions (with 61% of medical students in a recent national survey reporting experiencing weekly episodes of microaggressions)[7]

Copyright © 2022 Farid et al. This is an open-access publication distributed under the terms of the Creative Commons Attribution license.

to neglect,[8,3] inadequate student supervision, and perceptions of disrespect.[5]

In the OB/GYN clerkship in particular, students consistently report mistreatment, with up to one out of four clerkship students reporting mistreatment in a longitudinal study of over 800 students at one institution.[9] A smaller study with 18 students similarly found that 25% reported experiencing mistreatment on their OB/GYN rotation.[5] Baecher-Lind and colleagues hypothesized that in stressful clinical environments with high acuity, such as labor and delivery, communication breakdown contributes to feelings of perceived disrespect and neglect and missed educational opportunities.[5] In particular, labor and delivery encompass a variety of patients in different care settings, from a triage area serving as the obstetric emergency room to an inpatient unit with increasingly complex patients to an operating room handling emergent deliveries. Teaching across this broad clinical spectrum carries with it a unique set of challenges due to the different skills needed in each arena. The challenges of procedural teaching, such as the complexity of patient cases and lack of continuity with preceptors,[10] may lead to multiple episodes of neglect or disrespect, which degrade interpersonal interactions and contribute to an adverse learning environment.[11]

At our institution, reports from end-of-course evaluations over several years demonstrated episodes of learner mistreatment. Nearly 25% of students witnessed the use of unprofessional or derogatory language, did not receive constructive feedback, and noted a lack of empathy from OB/GYNs. Seventeen percent of students experienced a lack of respect on their OB/GYN clerkship. When compared to other clerkships at the institution, the OB/GYN clerkship ratings were consistently lower, and our hospital's site ratings were lower than other clerkship sites for OB/GYN within the academic institution. When we examined resident data based on an ACGME survey,[12] faculty engagement was identified as an area of growth. These findings prompted the OB/GYN medical education committee (graduate medical education, undergraduate medical education, and department leadership) to commit to change.

The first step we undertook was to promote faculty involvement in changing the learning environment through a faculty development session, which was inspired by the 2018 Macy Foundation conference detailing faculty development interventions to improve the learning environment.[13] To address concerns about the learning environment in our OB/GYN clerkship, we created a faculty development workshop to increase awareness, promote discussion, and problem-solve about this topic.

Our workshop is grounded in the principles of adult learning theory that encourage learners to take responsibility for their learning and to have an active role in the learning experience.[14] We utilize the concept of communities of practice for our workshop. A community of practice is structured around a group of people who care about the same issues and interact with each other regularly to learn from each other and address issues together. Our workshop targets faculty in the OB/GYN department, many of whom work with each other on a regular basis and all of whom are impacted by these concerns around the learning environment. We capitalize on people's acknowledgment that they all face the same problem to encourage them to problem-solve together. A community of practice is thus created in this workshop by bringing people together explicitly for the purpose of learning with and from each other.[15] By having colleagues work through problems they identify, the workshop engages participants so that they feel invested in the process of finding solutions that will work for their group. Other workshops[16,17] have described how to train participants to identify mistreatment or have asked for identification of barriers to creating a positive learning environment.[18] Our workshop expands on this prior work by having participants identify mistreatment and develop strategies to combat it. Thus, this workshop adds to the literature by describing a strategy that encourages participant involvement in creating solutions, along with a commitment to follow through on those solutions. We base this strategy on data illustrating that adult learners who are allowed to choose their own actions demonstrate higher rates of positive change.[19] While the workshop and curriculum are focused on OB/GYN faculty, other areas of medicine, particularly family medicine and surgery, can easily adapt the curriculum to their own field.

Methods

This workshop was offered as part of our department's quarterly faculty development grand rounds series, which was open to all faculty, residents, students, and staff in the department. Attendance at grand rounds was mandatory for all faculty and residents. Due to the COVID-19 pandemic, this workshop was offered virtually in 2021. There were no prerequisites to attend the workshop. Although both residents and faculty attended grand rounds, only faculty participants were invited to complete an electronic preworkshop survey (Appendix A) by email.

Members of the department's medical education committee reviewed end-of-clerkship evaluations from the medical students from the prior 6 months. Developed by the medical school, this evaluation was based on the AAMC Medical School Graduation

Copyright © 2022 Farid et al. This is an open-access publication distributed under the terms of the Creative Commons Attribution license.

Questionnaire.[20] The medical education committee focused on the domains in which the clerkship was most deficient, based on feedback from the medical students. These domains included constructive feedback, respectful interactions with students, using professional language, conflict resolution, and showing empathy. The committee worked with a research scientist with expertise in qualitative research to create a 12-item preworkshop survey to determine faculty teaching skills and confidence in engaging with and involving medical students and residents in clinical care. The survey also queried demographics, including age, gender identity, race and ethnicity, and number of years at the institution. Faculty were asked to complete the survey via an email sent 3 days prior to the event and sent again 1 day prior to initial nonresponders.

The introductory portion of the 60-minute workshop consisted of a PowerPoint presentation (Appendix B) and occurred in a virtual large-group setting. The first half of the presentation consisted of a summary of the quarterly student clerkship learning environment survey, as well as mistreatment reports at the institutional and departmental levels. The second half of the presentation focused on effective strategies to achieve positive change in the learning environment. We discussed the effect of physician stress and burnout on effective teaching.[21] We discussed the importance of culture change and our ability to do so using clinical teaching strategies that help to shape our learning environment. These clinical teaching strategies were adapted from Chuang and colleagues, who developed the report on behalf of the Association of Professors of Gynecology and Obstetrics Undergraduate Medical Education Committee.[22] The strategies included creating a climate of humanism, recognizing and discussing seminal events, role modeling, actively engaging learners, being relevant and practical, and synthesizing multiple strategies. This introduction took approximately 15 minutes.

After the introduction, participants were divided into six discussion groups of approximately 10 people each, facilitated by an experienced educator within the department. These breakout groups were conducted via Zoom, and an administrator randomly assigned participants to one of the breakout groups at the conclusion of the introduction. All the facilitators had a formal role within graduate or undergraduate medical education and served on the medical education committee for the department. All the faculty facilitators had at least 5 years of clinical and teaching experience.

Each of the facilitators was assigned to cover one of four cases (Appendix C). These cases had been created by the medical education committee, members of which were also group facilitators for the workshop. The cases covered a range of behaviors by faculty, staff, and residents ranging from minor (passive neglect) to blatant (belittling, overt humiliation) and were loosely modeled on events that had occurred within the institution. The cases were based on the medical student learning environment data indicating areas for improvement. Each case focused on two of the clinical teaching strategies from Chuang and colleagues[22] and highlighted aspects of the hidden curriculum that were utilized as prompts for further discussion. After initial development of the cases, six of the medical education committee members revised them to ensure that each case would meet the teaching objectives and be aligned with two of the clinical teaching strategies. The committee members matched the teaching strategies by identifying the most significant aspects of mistreatment in a case and connecting these to positive behaviors that could have been implemented instead. The cases were reviewed by the entire committee prior to finalization. The medical education committee also created a facilitator guide with questions to guide the discussion (Appendix D).

Each group chose a member to be the reporter. The groups read the assigned case, and the facilitator then led discussion through a series of questions focused on the related key domains. Facilitators encouraged participants to develop their own strategies to address specific aspects of the learning environment they found challenging. Discussion focused specifically on eliciting examples that promoted a positive learning environment. Breakout groups were allocated 25-30 minutes for reviewing and discussing the cases.

After that time, the administrator closed the breakout rooms, and all groups then reconvened for a report-out, with a group debriefing and discussion. A total of 15 minutes was allocated for this portion of the workshop. As participants reported out, one facilitator noted specific strategies for each aspect of the learning environment in a table (Appendix D) that was later shared via email with participants at the conclusion of the workshop. Each participant was asked to commit to using one strategy described by their fellow participants in the coming months.

Immediately after the workshop concluded, the faculty participants received a postworkshop survey sent via email (Appendix E). A reminder to complete the survey was sent a week later. The postworkshop survey included all the questions in the preworkshop survey and added three new ones: whether the participant's interactions with medical students and residents would change based on the workshop, the relevance of the workshop, and whether the participant had learned new skills

Copyright © 2022 Farid et al. This is an open-access publication distributed under the terms of the Creative Commons Attribution license.

in the workshop. A research assistant collated the open-ended responses and analyzed the quantitative data with simple descriptive statistics. After the workshop, the medical education committee also reviewed data from the learning environment questionnaire, which the medical school distributed to the next set of students completing the OB/GYN clerkship, to evaluate any changes in clerkship ratings.

Data were presented either as median with interquartile range or as proportion. Categorical data were compared using the chi-square or Fisher's exact test, whereas continuous data were compared using the Wilcoxon rank sum test. McNemar's test was used to calculate statistical differences between paired proportions. We considered *p* values less than .05 statistically significant. Data were analyzed with SAS 9.4 (SAS Institute). Institutional review board approval was granted as an exempt application.

Results

Sixty faculty members attended the workshop, representing the range of specialties in OB/GYN; 57% completed the preworkshop survey, and 33% completed the postworkshop survey (Table 1). The median age of the participants was 50, and the median number of years at the institution was 6. Three-quarters of the participants were female, and 50% were White. Eighteen residents participated in the workshop but did not receive the survey; medical students were not present during the workshop.

Prior to the session, while the majority (94%) of faculty who responded to the survey believed that they contributed to the learning environment, fewer (76%) felt they had the skills needed to be an effective teacher, and only 65% reported consistently trying to use effective teaching strategies. The vast majority (97%) aimed to create an inclusive environment, but only 68% of faculty reported they routinely involved medical students in the clinical care of patients. Only 62% felt prepared to engage medical students when they were the assigned faculty preceptor, although 76% felt prepared to engage residents as the assigned faculty preceptor. After the session, the majority of participants (75%) reported that they would consistently try to use effective teaching strategies (Table 2).

The majority of participants (85%) reported that after the workshop, they were more aware of issues around the learning environment. Most participants (85%) felt that their interactions with medical students would change in a positive way because of the workshop and the discussions it generated. Similarly, 80% of participants felt that their interactions with residents would change in a positive way because of the workshop, and they committed to creating an inclusive learning environment after the workshop (Table 3). However, none of these changes were statistically significant.

One area in which there were significant differences was faculty's self-rated ability to engage learners of different levels. When comparing faculty's self-perceived ability to engage residents versus students, the difference was statistically significant ($p = .014$). After the workshop, we found that faculty more frequently felt well prepared to engage both medical students (75%) and residents (85%), although they still were more likely to engage residents (Table 2); this difference was statistically significant ($p = .046$). There was also a statistically significant difference when we compared faculty's involvement of medical students versus residents specifically in clinical care. Faculty involved medical students in clinical care only 60% of the time, compared to 90% of the time for residents ($p = .01$; Table 2).

Table 1. Survey Respondent Demographics

	Presurvey ($N = 34$)		Postsurvey ($N = 20$)	
**Demographic**	***Mdn*** **(IQR)**	**No. (%)**	***Mdn*** **(IQR)**	**No. (%)**
Age	48 (40-47)		50 (41-58)	
Gender				
Female		26 (76)		15 (75)
Male		6 (18)		2 (10)
Prefer not to say		2 (6)		3 (15)
Race and ethnicity				
American Indian/Pacific Islander		1 (3)		0 (0)
Asian		4 (12)		3 (15)
Black/African American		2 (6)		2 (10)
Hispanic		4 (12)		2 (10)
White/Caucasian		20 (59)		10 (50)
Other		0 (0)		0 (0)
Prefer not to disclose		2 (6)		1 (5)
Years at the institution	8 (5-22)		6 (4-11)	

Abbreviation: IQR, interquartile range.

Copyright © 2022 Farid et al. This is an open-access publication distributed under the terms of the Creative Commons Attribution license.

Table 2. Understanding the Learning Environment

Faculty Attitudes	No. (%)	
	Presession ($N = 34$)	Postsession ($N = 20$)
I believe that as a faculty member, I contribute to the learning environment.		
Strongly agree or agree	32 (94)	18 (90)
Neutral	2 (6)	1 (5)
Strongly disagree or disagree	0 (0)	0 (0)
NA	0 (0)	1 (5)
I feel well prepared to engage medical students when I am the assigned faculty preceptor.		
Strongly agree or agree	21 (62)	15 (75)
Neutral	8 (24)	3 (15)
Strongly disagree or disagree	5 (15)	2 (10)
NA	0 (0)	0 (0)
I feel well prepared to engage residents when I am the assigned faculty preceptor.		
Strongly agree or agree	26 (76)	17 (85)
Neutral	7 (21)	1 (5)
Strongly disagree or disagree	1 (3)	0 (0)
NA	0 (0)	2 (10)
I have the skills needed to be an effective teacher.		
Strongly agree or agree	26 (76)	16 (80)
Neutral	7 (21)	4 (20)
Strongly disagree or disagree	1 (3)	0 (0)
NA	0 (0)	0 (0)
I consistently try to use effective teaching strategies.		
Strongly agree or agree	22 (65)	15 (75)
Neutral	11 (32)	4 (20)
Strongly disagree or disagree	0 (0)	1 (5)
NA	1 (3)	0 (0)
I aim to create an inclusive environment when teaching.		
Strongly agree or agree	33 (97)	18 (90)
Neutral	1 (3)	2 (10)
Strongly disagree or disagree	0 (0)	0 (0)
NA	0 (0)	0 (0)
I routinely involve medical students in the clinical care of patients.		
Strongly agree or agree	23 (68)	12 (60)
Neutral	7 (21)	5 (25)
Strongly disagree or disagree	3 (9)	3 (15)
NA	1 (3)	0 (0)
I routinely involve residents in the clinical care of patients.		
Strongly agree or agree	31 (91)	18 (90)
Neutral	0 (0)	1 (5)
Strongly disagree or disagree	0 (0)	0 (0)
NA	3 (9)	1 (5)

Abbreviation: NA, not applicable.

We collected and collated the strategies that the smaller discussion groups shared with the entire workshop during report-out and arranged them by themes. At the end of the session, participants were invited to commit to using one or more of these strategies in the future. The strategies that participants committed to ranged from simple (asking the student's name) to more complex (e.g., reminding themselves to be patient and encouraging increased communication). These strategies are summarized in Table 4.

Review of the free-text responses to the postsession evaluation demonstrated that multiple respondents appreciated the session's interactivity. In particular, respondents mentioned that videoconferencing promoted small-group discussion. Nearly all the respondents (90%) rated the session as being relevant to their needs, and 70% reported learning a new pedagogical skill during the workshop (Table 3). One participant wrote, "We need to be reminded that in these incredibly stressful times, we must make space for effective teaching."

The next learning environment questionnaire administered by the medical school after the workshop demonstrated significant improvement, with 100% of students reporting that faculty used professional language, demonstrated empathy, and were respectful towards students. The majority of students (86%) reported receiving constructive feedback.

Discussion

The hidden curriculum of a learning environment contributes substantially to students' development of professional behavior,

Copyright © 2022 Farid et al. This is an open-access publication distributed under the terms of the Creative Commons Attribution license.

Table 3. Responses to Questions Appearing Only on the Postsession Evaluation (N = 20)

Faculty Attitudes	No. (%)
I am more aware of the issues around the learning environment after today's presentation.	
Strongly agree or agree	17 (85)
Neutral	2 (10)
Strongly disagree or disagree	0 (0)
NA	1 (5)
I feel that my interaction with medical students will change in a positive way based on today's discussion.	
Strongly agree or agree	17 (85)
Neutral	1 (5)
Strongly disagree or disagree	1 (5)
NA	1 (5)
I feel that my interaction with residents will change in a positive way based on today's discussion.	
Strongly agree or agree	16 (80)
Neutral	2 (10)
Strongly disagree or disagree	0 (0)
NA	2 (10)
I am more inclined to create an inclusive learning environment after today's presentation.	
Strongly agree or agree	16 (80)
Neutral	2 (10)
Strongly disagree or disagree	1 (5)
NA	1 (5)
This faculty development grand rounds was relevant to my needs.	
Strongly agree or agree	18 (90)
Neutral	1 (5)
Strongly disagree or disagree	0 (0)
NA	1 (5)
I learned a new skill during this grand rounds.	
Strongly agree or agree	14 (70)
Neutral	4 (20)
Strongly disagree or disagree	1 (5)
NA	1 (5)

Abbreviation: NA, not applicable.

medical knowledge, and clinical skills, and a positive learning environment has been demonstrated to support the acquisition of skills.[23] This workshop provides a framework to discuss the hidden curriculum and its impact on the learning environment, as well as strategies to improve the learning environment through case-based discussion among educators and faculty. We relied on the principles of adult learning theory to empower learners to take responsibility for their learning[14] and explicitly created a community of practice[15] to allow them to discuss shared experiences and devise solutions applicable to the group. We provided participants a framework with which to address aspects of the hidden curriculum, but the participants themselves arrived at suggestions to improve the learning environment in six key domains.[22]

This workshop was successful and relevant in part because the participants were from one department and knew each other well, thereby providing a safe environment among trusted colleagues and enabling active engagement and honest discussion. The scenarios were modeled on actual experiences and demonstrated aspects of the hidden curriculum (including microaggressions, neglect, and humiliation) that impact the learning environment. We would recommend that in the smaller breakout sessions, one person be designated at the very beginning to report out to the large group in order to facilitate their taking notes or preparing to share the group's discussion. Having breakout groups of approximately 10 participants was helpful because each group was small enough to encourage participation but large enough that, if a few people did not

Table 4. Commitment to Change

How Do You ...	Examples of Strategies
Establish a climate of humanism (create a supportive learning environment)?	Demonstrate that the patient is the center of your interactions.<br>Build a relationship with the patient.<br>Remember that medicine is about our shared humanity.
Role model (demonstrate skills)?	Ask the patient what they value.<br>Model professionalism in all your interactions with patients, colleagues, and staff.<br>Check in with your team; model care and compassion.<br>Give positive and meaningful feedback on what was done well.
Actively engage the learner (involve the learner)?	Emphasize communication to improve feeling of inclusion in the team and also to share responsibility.<br>Set expectations/goals at the beginning of an encounter.<br>Ask the learner: What would you like to learn?<br>Begin with introductions; get to know something about the student.<br>Create an environment where people can trust each other and assume best intentions.<br>Gradually give the students more independence; thank them for being interested and engaged.
Be practical and relevant (focus on small skills that are generalizable)?	Setting mutually agreed-upon learning objectives.<br>Have the student observe faculty with one patient encounter.<br>Set expectations for what part of the case/patient encounter the learner will participate in.<br>Have focused teaching topics.<br>Give specific feedback about what learners can continue to work on.
Recognize and use seminal events (when bad things happen)?	Debrief with all learners after adverse event.<br>Have open and honest conversations about systems-based mistakes.<br>Encourage open communication.<br>Discuss and model how to learn from a medical error.
Use ongoing and multiple strategies (put it all together)?	Meet the learner where they are.

Copyright © 2022 Farid et al. This is an open-access publication distributed under the terms of the Creative Commons Attribution license.

participate, the discussion was not negatively impacted. One hour was sufficient time for the workshop. Given the sensitive nature of the discussion, facilitators for the workshop should be experienced medical educators.

Multiple participants commented on the interactivity of the workshop; particularly when conferences are virtual, an interactive component is key.[24] Sharing educational strategies demonstrated a commitment to medical education by participants that other faculty and residents could emulate. While other curricula have described workshops discussing the learning environment, ours specifically provided participants with a framework to critically analyze the learning environment and then develop their own solutions using that framework. Asking participants to commit to using at least one strategy confirmed their dedication to addressing the learning environment and reinforced the need for change. Data from reviews of motivational interviewing, in which participants reach their own decisions about how to implement behavioral change, have demonstrated increased rates of positive change[19] and increased self-efficacy.[25] Extrapolating these data to our workshop, we anticipate that the commitments to change generated by the participants themselves will have some ongoing impact.

Although faculty felt more prepared to engage residents than students and included residents more often in clinical work both before and after the workshop, overall there was an upward trend for feeling better prepared when engaging medical students. Interestingly, while prior to the workshop 68% of faculty involved medical students in clinical care, immediately after the workshop 60% of faculty involved medical students in clinical care. We believe that these numbers do not represent a true decline in faculty's involvement of medical students but rather may be due to the fact that the number of faculty who completed the postworkshop survey was small. We acknowledge that faculty may find it easier to engage and teach residents who are in the program for 4 years, rather than students who have rotations lasting just a few weeks, with time fragmented between different services on the rotation. This lack of continuity with students may make it more challenging for faculty to feel invested in individual students and to expend their time teaching. In addition, some faculty may prefer or find it easier to teach learners who are already committed to the specialty rather than students who may still be exploring career interests. Within a procedural specialty, faculty may also be struggling to balance learners at different levels and may fear that focusing on one learner could detract from the learning opportunities of the others. The difference between faculty preparedness for teaching and inclusion in clinical care between students and residents remained statistically significant even after the workshop; these findings merit further research. We hope to include a future session with a focus on teaching to one's highest abilities with multiple levels of learners.

Limitations of this work included a small number of participants from a single department at one academic medical center. Our cases are directly relevant to OB/GYN, but other specialties could adapt the cases to reflect clinical examples from their field. Another limitation was a small response rate for the postworkshop survey; response rates could have been improved if time was allocated at the end of the session to complete the postworkshop survey, which took approximately 5 minutes. In addition, pre- and postsurveys were not matched to directly compare respondents' answers. If nonresponders are significantly less likely to have improved then responders, we may be overestimating the effect of our workshop.

Finally, we have limited long-term data regarding changes in the learning environment, thereby making it difficult to determine the long-term impact of the workshop, although we plan to continue tracking data pertaining to the learning environment. Since the workshop, initial updated data about the learning environment from our institution demonstrated an internal upward trend in 80% of the domains examined by the institution, as well as higher ratings in 100% of the domains when compared with the other sites at which medical students rotate. We will continue to track data for 12-18 months after the workshop to assess for long-term improvement.

In summary, this interactive, virtual workshop enabled a vital, guided discussion around the hidden curriculum and the learning environment and empowered participants to create their own strategies for how to improve the learning environment. The workshop encouraged participants to commit to one teaching strategy to address deficits in the learning environment, which served to strengthen their commitment to medical education. Limited data collected from our institution demonstrated improvement in the learning environment and an upward trend for our hospital in particular when compared to the other sites. The learning environment is intertwined with students' academic success and personal well-being. Engaging the faculty who help shape that learning environment is crucial if there is to be impactful, effective change that will help maximize our learners' success. As we train the next generation of physicians, we have an obligation to our patients and to society to ensure that the milieu in which they learn how to care for others is supportive, encouraging, and fulfilling.

Copyright © 2022 Farid et al. This is an open-access publication distributed under the terms of the Creative Commons Attribution license.

Appendices

A. Preworkshop Survey.docx

B. PowerPoint for the Learning Environment.pptx

C. Cases for the Learning Environment.docx

D. Facilitator Guide.docx

E. Postworkshop Survey.docx

All appendices are peer reviewed as integral parts of the Original Publication.

Huma Farid, MD: Instructor, Department of Obstetrics, Gynecology, and Reproductive Biology, Beth Israel Deaconess Medical Center, Harvard Medical School; ORCID: https://orcid.org/0000-0001-8041-4251

John L. Dalrymple, MD: Professor, Department of Obstetrics, Gynecology, and Reproductive Biology, Beth Israel Deaconess Medical Center, Harvard Medical School

Monica Mendiola, MD: Instructor, Department of Obstetrics, Gynecology, and Reproductive Biology, Beth Israel Deaconess Medical Center, Harvard Medical School

Celeste Royce, MD: Assistant Professor, Department of Obstetrics, Gynecology, and Reproductive Biology, Beth Israel Deaconess Medical Center, Harvard Medical School

Brett Young, MD: Assistant Professor, Department of Obstetrics, Gynecology, and Reproductive Biology, Beth Israel Deaconess Medical Center, Harvard Medical School

Katharyn Meredith Atkins, MD: Assistant Professor, Department of Obstetrics, Gynecology, and Reproductive Biology, Beth Israel Deaconess Medical Center, Harvard Medical Schools

Disclosures
None to report.

Funding/Support
None to report.

Ethical Approval
The Beth Israel Deaconess Medical Center Institutional Review Board deemed further review of this project not necessary.

References

1. Genn JM. AMEE Medical Education Guide no. 23 (part 1): curriculum, environment, climate, quality and change in medical education—a unifying perspective. *Med Teach*. 2001;23(4): 337-344. https://doi.org/10.1080/01421590120063330
2. Hafferty FW. Beyond curriculum reform: confronting medicine's hidden curriculum. *Acad Med*. 1998;73(4):403-407. https://doi.org/10.1097/00001888-199804000-00013
3. Castillo-Angeles M, Watkins AA, Acosta D, et al. Mistreatment and the learning environment for medical students on general surgery clerkship rotations: what do key stakeholders think? *Am J Surg*. 2017;213(2):307-312. https://doi.org/10.1016/j.amjsurg.2016.10.013
4. Pai PG, Menezes V, Srikanth, Subramanian AM, Shenoy JP. Medical students' perception of their educational environment. *J Clin Diagn Res*. 2014;8(1):103-107. https://doi.org/10.7860/JCDR/2014/5559.3944
5. Baecher-Lind LE, Chang K, Blanco MA. The learning environment in the obstetrics and gynecology clerkship: an exploratory study of students' perceptions before and after the clerkship. *Med Educ Online*. 2015;20:27273. https://doi.org/10.3402/meo.v20.27273
6. Cook AF, Arora VM, Rasinski KA, Curlin FA, Yoon JD. The prevalence of medical student mistreatment and its association with burnout. *Acad Med*. 2014;89(5):749-754. https://doi.org/10.1097/ACM.0000000000000204
7. Anderson N, Lett E, Asabor EN, et al. The association of microaggressions with depressive symptoms and institutional satisfaction among a national cohort of medical students. *J Gen Intern Med*. 2022;37(2):298-307. https://doi.org/10.1007/s11606-021-06786-6
8. Buery-Joyner SD, Ryan MS, Santen SA, Borda A, Webb T, Cheifetz C. Beyond mistreatment: learner neglect in the clinical teaching environment. *Med Teach*. 2019;41(8):949-955. https://doi.org/10.1080/0142159X.2019.1602254
9. Oser TK, Haidet P, Lewis PR, Mauger DT, Gingrich DL, Leong SL. Frequency and negative impact of medical student mistreatment based on specialty choice: a longitudinal study. *Acad Med*. 2014;89(5):755-761. https://doi.org/10.1097/ACM.0000000000000207
10. Grantcharov TP, Reznick RK. Teaching procedural skills. *BMJ*. 2008;336(7653):1129-1131. https://doi.org/10.1136/bmj.39517.686956.47
11. Nordquist J, Hall J, Caverzagie K, et al. The clinical learning environment. *Med Teach*. 2019;41(4):366-372. https://doi.org/10.1080/0142159X.2019.1566601
12. ACGME resident/fellow survey content areas. Accreditation Council for Graduate Medical Education. November 2019. Accessed March 31, 2022. https://www.acgme.org/globalassets/ResidentSurvey_ContentAreas.pdf
13. Irby DM. *Improving Environments for Learning in the Health Professions: Proceedings of a Conference Sponsored by Josiah Macy Jr. Foundation in April 2018*. Josiah Macy Jr. Foundation; 2018.
14. Spencer JA, Jordan RK. Learner centred approaches in medical education. *BMJ*. 1999;318(7193):1280-1283. https://doi.org/10.1136/bmj.318.7193.1280

Copyright © 2022 Farid et al. This is an open-access publication distributed under the terms of the Creative Commons Attribution license.

15. Pyrko I, Dörfler V, Eden C. Thinking together: what makes communities of practice work? *Hum Relat*. 2017;70(4):389-409. https://doi.org/10.1177/0018726716661040

16. Lewis J, Feldman N, Rich A, Ackerman S, Patel C. Positive learning environment and mistreatment prevention module. *MedEdPORTAL*. 2015:11;10113. https://doi.org/10.15766/mep_2374-8265.10113

17. Fleit HB, Lu WH, Olvet DM, Chandran L. Case studies for recognizing appropriate and inappropriate behaviors in the clinical learning environment. *MedEdPORTAL*. 2017:13;10638. https://doi.org/10.15766/mep_2374-8265.10638

18. Rich A, Ackerman S, Patel C, Feldman N, Adams D, Lewis J. Creating a positive learning environment: educational film and discussion guide. *MedEdPORTAL*. 2015:11;10131. https://doi.org/10.15766/mep_2374-8265.10131

19. Burke BL, Arkowitz H, Menchola M. The efficacy of motivational interviewing: a meta-analysis of controlled clinical trials. *J Consult Clin Psychol*. 2003;71(5):843-861. https://doi.org/10.1037/0022-006X.71.5.843

20. Graduation Questionnaire (GQ). Association of American Medical Colleges. 2022. Accessed March 31, 2022. https://www.aamc.org/data-reports/students-residents/report/graduation-questionnaire-gq

21. Burnout an “occupational phenomenon”: International Classification of Diseases. World Health Organization. May 28, 2019. Accessed March 31, 2022. https://www.who.int/news/item/28-05-2019-burn-out-an-occupational-phenomenon-international-classification-of-diseases

22. Chuang AW, Nuthalapaty FS, Casey PM, et al. To the point: reviews in medical education—taking control of the hidden curriculum. *Am J Obstet Gynecol*. 2010;203(4):316.e1-316.e6. https://doi.org/10.1016/j.ajog.2010.04.035

23. Hoff TJ, Pohl H, Bartfield J. Creating a learning environment to produce competent residents: the roles of culture and context. *Acad Med*. 2004;79(6):532-540. https://doi.org/10.1097/00001888-200406000-00007

24. Rubinger L, Gazendam A, Ekhtiari S, et al. Maximizing virtual meetings and conferences: a review of best practices. *Int Orthop*. 2020;44(8):1461-1466. https://doi.org/10.1007/s00264-020-04615-9

25. Evans DS, Martin L, Neeson B, O’Brien M, Cahill D. *Brief Interventions and Motivational Interviewing: Literature Review and Guidance for Practice*. Health Service Executive; 2011. http://hdl.handle.net/10147/204231

Received: October 22, 2021
Accepted: February 8, 2022
Published: May 3, 2022

Copyright © 2022 Farid et al. This is an open-access publication distributed under the terms of the Creative Commons Attribution license.
